# Frequency of Phytoestrogen Consumption and Symptoms at Midlife among Bangladeshis in Bangladesh and London

**DOI:** 10.3390/nu15173676

**Published:** 2023-08-22

**Authors:** Lynnette Leidy Sievert, Taniya Sharmeen, Khurshida Begum, Shanthi Muttukrishna, Osul Chowdhury, Gillian R. Bentley

**Affiliations:** 1 Department of Anthropology, UMass Amherst, Amherst, MA 01003, USA; 2 Nuffield Department of Medicine, University of Oxford, Oxford OX3 7BN, UK; 3 Department of Anthropology, Durham University, Durham DH1 3LE, UK; 4 Department of Obstetrics and Gynecology, University College Cork, T12 E7WX Cork, Ireland; 5 Parkview Medical College, Sylhet 3100, Bangladesh

**Keywords:** menopause, hot flashes, vaginal dryness, phytoestrogens, lignans

## Abstract

There is a longstanding interest in the relationship between diet and hot flash symptoms during midlife, especially in whether phytoestrogens ease menopausal symptoms. The purpose of this study was to examine hot flashes, night sweats, trouble sleeping, and vaginal dryness in relation to the intake of foods rich in phytoestrogens among Bangladeshi women aged 35 to 59 years who were living either in Sylhet, Bangladesh (*n* = 157) or as migrants in London (*n* = 174). Consumption ranges for phytoestrogens were constructed from food frequencies. We hypothesized that diets rich in isoflavones, lignans, and coumestrol would be associated with lower symptom frequencies. However, adjusted logistic regression results showed that with each incremental increase in general phytoestrogen consumption (scale of 0 to 10), the likelihood of hot flashes increased by 1.4%. Each incremental increase in lignan consumption raised the likelihood of hot flashes by 1.6%. In contrast, the odds of vaginal dryness decreased by 2%, with each incremental increase in phytoestrogen and lignan consumption, and by 4%, with each incremental increase in isoflavone consumption. Night sweats and trouble sleeping were not associated with phytoestrogen intake in logistic regressions. Our findings add to the conflicting data on relationships between phytoestrogens and symptoms associated with menopause.

## 1. Introduction

There is a longstanding interest in the relationship between diet and hot flash symptoms at midlife prompted initially by observations that Japanese women, who eat a diet rich in phytoestrogens [1,2], reported lower frequencies of hot flashes compared to women of European descent [3,4,5,6,7,8,9]. In the baseline Study of Women’s Health Across the Nation, only 12% of Japanese Americans reported hot flashes within the past two weeks compared to 26% of women of European descent, 26% of Latinas, and 39% of Black women [10].

Phytoestrogens are compounds that are structurally similar to estrogens. At menopause, when a woman’s own estrogen levels are low, foods rich in phytoestrogens can have estrogenic effects [11]. A low level of estrogen at menopause is the context for the neuroendocrine triggers of hot flashes [12,13,14]. Therefore, a higher intake of phytoestrogenic foods may reduce menopausal symptoms.

The purpose of the investigation presented here was to examine four of the most common symptoms at midlife (specifically, hot flashes, night sweats, trouble sleeping, and vaginal dryness) in relation to the intake of foods rich in phytoestrogens among Bangladeshi women living either in London or Sylhet, Bangladesh. We consider these symptoms in the context of the food culture of Bangladeshi women and how dietary choices may contribute to symptoms associated with menopause.

Phytoestrogens include three major classes: isoflavones, lignans, and coumestans. Isoflavones (including genistein, daidzein, and glycitein) are found in soybeans and other legumes and have been investigated in relation to menopausal symptoms, cardiovascular health, osteoporosis, and hormone-dependent cancers [15,16,17]. Among isoflavones, daidzein can be metabolized by gut bacteria to produce equol, which has a chemical structure similar to that of estrogen and, thus, equol can bind to estrogen receptors [18,19]. People following vegetarian diets appear to produce equol more frequently compared to omnivores [18,20]. Variation in the ability to make equol may explain some inter-individual variation in the frequency of hot flashes, with equol production or supplementation alleviating hot flashes [21,22].

Lignan precursors are found in most plants but primarily in flaxseed, legumes, fruits, vegetables, and whole grains [23,24]. Most, but not all [25], studies have shown no association between lignan supplementation and hot flashes [26,27,28,29,30]. Foods high in coumestrol, a type of coumestan, include alfalfa and clover sprouts, split peas, and pinto beans. In addition, a type of chickpea eaten in Bangladesh called *kala chana* is high in coumestrol [31]. In a meta-analysis of five studies, there was no evidence of a decrease in hot flash incidence among participants taking a red clover extract [32].

Randomized trials of soy and other phytoestrogens in relation to hot flash experiences have been carried out for decades [33,34,35,36,37]. Most trials supplement or substitute phytoestrogenic nutrients into everyday diets. For example, in a recent study, women aged 42 to 64 years were assigned to either a low-fat, vegan diet that included half a cup of cooked soybeans daily or no dietary change for 12 weeks. Self-reported hot flashes decreased in both groups but more so in the intervention group [38].

In addition to studies of phytoestrogen supplementation, hot flashes have been examined in relation to overall diet as assessed by food frequency questionnaires. Food combinations reflect food preferences as well as cultural, social, environmental, and economic determinants [39]. Among peri-menopausal women in the U.S., vegans reported significantly less bothersome vasomotor symptoms compared to omnivores, and total vegetable intake was associated with a decrease in symptoms [40]. The longitudinal Women’s Health Initiative Dietary Modification trial in the U.S. found that dietary modification reduced the likelihood of vasomotor symptoms beyond the effect of weight loss [41]. In the Australian Longitudinal Study, six patterns of dietary intake were identified, and fruit intake and Mediterranean-style diets were associated with lower odds of vasomotor symptoms [42]. Among postmenopausal Chinese women in Hong Kong aged 48 to 70 years, whole-plant food scores were negatively associated with the odds of non-vasomotor symptoms; however, whole-plant food did not significantly lower the odds of hot flashes [39].

For the research presented here, we made use of food frequencies collected as part of a study of reproductive aging [43,44] and symptoms at midlife [45,46,47] among first-generation Bangladeshi immigrants living in London, UK and Bangladeshis living in Sylhet, Bangladesh (sedentees). Seventy-seven percent of the women in Sylhet and all of the Bangladeshi immigrants in our London sample were Muslim, which influences food choices. For example, there is a sharp seasonal increase in the intake of chickpeas (boot/garbanzo/*kala chana*), chickpea flour, and Bengal gram (chola) during the holy month of Ramadan (a month-long period of fasting). For this study, our food list was specific to the vegetables and spices commonly eaten by Bangladeshis throughout the year. Soy is not a food commonly eaten in Bangladesh or among Bangladeshi immigrants in London.

The purpose of this investigation was to examine symptoms at midlife in relation to the diet of Bangladeshi women living in London and Sylhet, Bangladesh. We hypothesized that diets with vegetables rich in phytoestrogens (isoflavones, lignans, and coumestrol) would be associated with a lower likelihood of hot flashes, night sweats, trouble sleeping, and vaginal dryness. In contrast to most studies of phytoestrogens and the menopausal transition, we included other symptoms potentially related to low estrogen levels. Both hot flashes and night sweats are often studied together as vasomotor symptoms associated with the menopausal transition. Trouble sleeping has multifactorial causation but has been shown to be associated with menopausal stages and may be related to mood disorders, hot flashes, or night sweats [48,49]. Finally, as estrogen levels fall, vaginal dryness worsens during and after the menopausal transition [50].

## 2. Methods

Data were drawn from a larger study of reproductive aging and symptom experience carried out in 2006–2010 among women aged 35 to 59 years residing in Sylhet, Bangladesh (sedentees, *n* = 157) and Bangladeshi immigrants who were born in Bangladesh and moved to London as adults (*n* = 174). We included women as young as 35 because of the earlier age at menopause in Bangladesh. In this study, mean recalled ages at menopause were 45.8 years among postmenopausal sedentees and 47.5 years among postmenopausal Bangladeshi migrants [44]. We expected symptoms to start at a relatively early age in this population because the hormonal changes associated with menopause started at an early age [43].

Exclusion criteria included history of hysterectomy, use of hormone therapy, current pregnancy, lactation, or use of hormonal contraceptives. The larger study included London women of European descent (*n* = 154) who lived in neighborhoods similar to the Bangladeshi immigrants; however, those participants are not included in the analyses here because the food list was developed to capture phytoestrogen intake specifically among Bangladeshi women.

Bangladeshi women were recruited from the general community in Sylhet with the help of undergraduate students from Shahjalal University and influential community members. In London, Bangladeshi women were recruited from community centers with the help of local contacts and through advertisements in free local papers. The majority of participants came from the borough of Camden.

In all sites, women participated in face-to-face interviews with semi-structured questionnaires and anthropometric measurements (height, weight, waist, and hip circumferences). Interviews and anthropometric measures were carried out in Bengali (the national language of Bangladesh) or Sylheti (the local dialect in Sylhet) by two authors (K.B. and T.S.). The questionnaires collected demographic, reproductive, and biobehavioral information relevant to age at menopause and symptom experience at midlife. In Bangladesh, we piloted the questionnaire for comprehensibility among women in the capital city of Dhaka using standard Bengali before moving to Sylhet. In Sylhet, the questionnaire was translated to the Sylheti dialect and back-translated to check accuracy of meaning with help from colleagues at Shahjalal University.

### 2.1. Food List

There is limited variation in the types of vegetables consumed in Bangladesh [51], and commonly used vegetables are grown in every part of the country. Bangladeshi migrants to the UK come primarily from Sylhet City and Division [52,53], and immigrants retain and maintain food choices in the UK similar to their home country [54]. In London, Bangladeshi foods are imported for consumption and are available in Bangladeshi neighborhoods. In addition, hyacinth beans (*Lablab purpureus*), known as *sheem* or *uri,* are common plants in Bengali gardens in London and are grown from seeds transported from Bangladesh [54]. The hyacinth bean is a close relative of the common bean, and the flavonoids genistein, 2-hydroxygenistein, dalbergioiden, and isoflavone kievitone are found in hyacinth bean hypocotyls [55].

Seeds of the hyacinth plant are cooked fresh while in season or, when fresh beans are out of season, eaten dry roasted as a snack or cooked as a curry with fish throughout the year. Nuts and seeds are usually dry roasted and consumed as snacks, e.g., peanuts, almonds, cashews, pumpkin seeds, jackfruit seeds, lotus seeds, and mustard seeds. Mustard seeds are also ground into a paste for curries used almost daily in Hindu households in Sylhet. In Muslim households, most curry bases are made of onion, garlic, and ginger paste with spices, but in Hindu households, a mix of nigella seeds and mustard paste is used instead of onions and garlic. Chickpeas, Bengal gram, and yellow peas are also commonly consumed as dry roasted snacks and as cooked snacks (e.g., *chotpoti* and *chola bhaji*).

We constructed our food list by focusing on food items commonly consumed in Sylheti households. We asked, “Which of the following do you eat and how often?” followed by a list of 33 foods. We asked whether each food was eaten raw or cooked and the frequency of consumption (daily, weekly, fortnightly, monthly, occasionally, or never). We did not query the amount of food eaten. After piloting the questionnaire in Dhaka, we added the following to our original list: sweets (e.g., *halwa*, *laddu*) made of chickpea flour (gram flour or *besan*), dry roasted bean kernels (chickpea, yellow split pea, hyacinth bean, or soybean), and snacks made out of chickpea flour or ground red lentils (e.g., *pakoras*, *bhajis*).

### 2.2. Symptom List

The symptom list was developed from a list of everyday complaints used by menopause researchers in Japan, Canada, the U.S., Australia, Lebanon, Morocco, Spain, Mexico, and other countries [4,56,57,58]. Some additional symptoms from the Greene Climacteric Index were added [59]. The question was, “Thinking back over the past two weeks, have you ever been bothered by any of the following?” This was followed by 26 symptoms early in the questionnaire and by seven additional culturally sensitive symptoms (e.g., loss of sexual desire, vaginal dryness) later in the questionnaire. The latter, sensitive questions were not asked among unmarried Bangladeshi women. For this reason, analyses of vaginal dryness (below) have smaller sample sizes. Women answered with “not at all”, “a little”, “quite a bit”, or “extremely”. For this study, symptoms were collapsed into bivariate yes/no categories.

### 2.3. Covariates

Risk factors for hot flashes include menopausal status, smoking, alcohol consumption, low socioeconomic status, and high levels of stress [60,61,62]. The relationship between hot flashes and BMI is complicated by whether women are early in the menopausal transition (heavier women are more likely to have hot flashes) or later in the menopausal transition (leaner women are more likely to have hot flashes) [63]. Moreover, in this population, clothing, and activities related to Muslim and Hindu religions were associated with the experience of hot flashes [47,64]

Menopausal status was categorized as premenopausal (regular menstruation or small changes in frequency or amount of bleeding), peri-menopausal (irregular menstruation or missing entire months of menstruation), and postmenopausal (no menstruation for 12 months or more.) Bangladeshi women do not smoke cigarettes (only one migrant smoked in this sample), but over a third of the women in this sample (38%) used tobacco in betel nut quid, which is frequently chewed among older Bangladeshi women [65]. Twelve participants did not answer the question about smoking or use of betel nut. Bangladesh has an almost zero level of alcohol consumption [66], and only one migrant reported drinking alcohol in this sample; therefore, alcohol use was not included in the study. Financial comfort was measured by the question, “In terms of your current financial situation are you struggling, OK, comfortable, or well-off?” Two participants did not answer this question. Stress was measured by the question, “In the past year, how would you describe the amount of stress in your life, on a scale of 1–6 (1 no, 6 extreme)?” Thirteen women did not answer this particular question. BMI was computed as weight divided by height^2^ (kg/m^2^). Muslim (90%) and Hindu (10%) women were included in analyses, while the one Christian was excluded. Missing data for covariates contributed to variation in sample sizes in the analyses below.

### 2.4. Analyses

We created consumption range values for phytoestrogen consumption by estimating the average intake of foods per month. Monthly intake was assigned a value of 1 and a continuous scale was created: never = 0, occasionally = 0.33, monthly = 1, fortnightly = 2, weekly = 4, between daily and weekly = 7, and daily = 10. The continuous values (0 to 10) were used to estimate the overall intake of phytoestrogens as the sum of frequencies for each of the individual foods in the category.

We created four consumption ranges based on published levels of phytoestrogens, isoflavones, lignans, and coumestan (Table 1). The isoflavone, lignan, and coumestan consumption ranges are subsets of the phytoestrogen consumption range. (1) The phytoestrogen consumption range was estimated from the intake of tomatoes + red lentils + cauliflower + cabbage + carrots + broad beans + cucumbers + pumpkin + mung beans + nuts and seeds + dried peas + dried hyacinth beans + chickpeas + garbanzo beans + chickpea flour + broccoli. (2) The lignan consumption range was estimated from the intake of tomatoes + cauliflower + cabbage + carrots + broad beans + cucumbers + pumpkin + mung beans + nuts and seeds + dried hyacinth beans + chickpeas + garbanzo beans + chickpea flour + broccoli. (3) The isoflavone consumption range was estimated from the intake of red lentils + mung beans + nuts and seeds + dried peas + dried hyacinth beans + chickpeas + garbanzo beans + chickpea flour. (4) The coumestan consumption range was estimated from the intake of dried peas + chickpeas. We did not ask separately about *kala chana*, but some of the chickpeas may be of this type.

Symptoms associated with declining levels of estrogen, specifically hot flashes, night sweats, trouble sleeping, and vaginal dryness (yes/no) were examined in relation to variables selected on the basis of the literature and previous findings. Bivariate analyses were used to determine which variables to include in logistic regression analyses for each symptom. Chi-square tests were carried out to examine symptoms in relation to migrant status (sedentee/ migrant), menopausal status (pre, peri, post), marital status (yes/no), parity (0, 1–2, 3–4, 5+), financial comfort (struggling, OK, comfortable, well-off), religion (Muslim/Hindu), and smoke/use tobacco with betel nut (yes/no). T-tests were used to examine BMI (kg/m^2^), stress (scale of 0–6), and the four dietary consumption ranges in relation to symptoms (yes/no).

Each symptom was examined with logistic regression analyses in relation to menopausal status, dietary consumption ranges (in separate models because of overlap among the foods included), and variables significant at *p* < 0.05 in the bivariate analyses. Marital status was not associated with any of the symptoms at *p* < 0.05 and was not included in any logistic regression. Models were carried out for all Bangladeshis (migrants and sedentees combined), for London migrants alone, and for sedentees in Sylhet, Bangladesh alone.

### 2.5. Ethics

Ethical approval was obtained from the Institutional Review Board of UMass Amherst, award number 004081, and Ethics Committees at University College London, Durham University, and the M.A.G. Osmani Medical College, Sylhet, Bangladesh. The consent document was explained in Bengali or Sylheti.

## 3. Results

Sample characteristics are shown in Table 2. Bangladeshi sedentees and migrants did not differ by age, menopausal status, or marital status, but migrants had more children. Sedentees were more likely to describe themselves as “well-off” compared to migrants. This is because we targeted Bangladeshis in Sylhet who had the means to emigrate to London in order to make comparisons with London migrants. Once migrants arrive in the UK, even if highly educated, they may not be able to find comparable jobs, and currency exchange rates are not equivalent. Therefore, migrants were more likely to describe themselves as financially “struggling” (Table 2). Compared to migrants, sedentees were thinner, reported a lower level of stress, and consumed significantly more phytoestrogens. There was a higher proportion of Hindus among the sedentees. In general, Hindus are more likely to be vegetarian, although we did not specifically ask this question.

The most frequently consumed foods among sedentees and migrants included tomatoes, red lentils, cauliflower, cucumbers, carrots, broad beans, and cabbage. These were most often eaten daily or weekly (Table 1). Intake of particular foods differed between sedentees and migrants; for example, 52% of sedentees reported eating red lentils every day compared to 14% of migrants. Sedentees were also more likely to eat tomatoes, cauliflower, carrots, and broad beans every day compared to migrants. On the other hand, migrants were more likely to eat garbanzo beans (chola) at least occasionally (70%) compared to sedentees (57%).

From the bivariate analyses used to determine which variables to include in the logistic regressions for symptom occurrence, significant associations were seen, as expected, between menopausal status and hot flashes, night sweats, trouble sleeping, and vaginal dryness (Supplemental Tables S1 and S2). Parity was associated with hot flashes, night sweats, and trouble sleeping. Financial comfort and use of tobacco were associated with night sweats and trouble sleeping. Trouble sleeping was also associated with BMI and stress.

In bivariate *t*-test results, contrary to our expectations, women with hot flashes during the past two weeks ate foods rich in lignans more often compared to women without hot flashes (*p* < 0.05; Table 3). On the other hand, consistent with our expectations, women who reported trouble sleeping consumed phytoestrogens, lignans, and isoflavone-containing foods less often than women without trouble sleeping (*p* < 0.05). Finally, women who reported vaginal dryness consumed foods rich in lignans and isoflavones less often than women without vaginal dryness (*p* < 0.05).

Logistic regression results indicated that both the estimated frequency of phytoestrogen intake and the estimated frequency of lignan intake were significantly associated with hot flashes in the total sample (Table 4); however, the direction of the relationships did not meet our initial expectations. After adjusting for menopausal status and parity, the odds of hot flashes increased by 1.4% with each incremental increase in the phytoestrogen consumption range, and the odds of hot flashes increased by 1.6% with each incremental increase in the lignan consumption range. The significant relationships between hot flashes and phytoestrogen and lignan consumption remained significant in the model limited to sedentees (Table 4), and the direction of the relationship was the same in the model limited to London migrants. Isoflavone and coumestrol consumption were not associated with hot flashes (models not shown). In addition, and as expected, compared to premenopausal women, peri-menopausal women had four times the odds of reporting hot flashes in the total sample.

Consistent with the bivariate results, none of the estimates for the frequency of phytoestrogen intake was associated with night sweats in logistic regressions in the total sample or in models limited to sedentees or London migrants (Table 5). However, Table 5 shows that, with the addition of each child, there was about a 20% increase in the odds of night sweats, and compared to women who struggle financially, the odds of night sweats decreased substantially as women described their financial situation as OK, comfortable, or well off. Hindu participants were four times more likely to report night sweats compared to Muslim participants in Sylhet, Bangladesh.

After adjusting for covariates, none of the estimates of the frequency of phytoestrogen intake was associated with trouble sleeping (Table 6). Logistic regression models showed that postmenopausal women had more than twice the odds of trouble sleeping compared to premenopausal women. This relationship remained significant in the model with migrants alone but not in the model with sedentees. Self-reported stress increased the odds of trouble sleeping in the sample as a whole and also when migrants were considered alone. Using tobacco more than doubled the odds of trouble sleeping in the total sample. This relationship remained significant in the model with only sedentees. For each incremental increase in BMI, the odds of trouble sleeping increased by 10%. This relationship also remained significant in the model with only sedentees (Table 6).

Finally, after adjusting for menopausal status, the likelihood of vaginal dryness declined with phytoestrogen intake so that, with each incremental increase in the frequency of phytoestrogen or lignan intake, there was a decline of about 2% in the odds of vaginal dryness. With each incremental increase in the frequency of isoflavone intake, there was a decline of about 4% in the odds of vaginal dryness (Table 7). There was no relationship between vaginal dryness and coumestan consumption. These consistent relationships did not remain significant in the models with only migrants and with only sedentees, although the direction of the relationships remained the same. Compared to premenopausal women, peri-menopausal women had more than three times the odds, and postmenopausal women had more than five times the odds of vaginal dryness.

## 4. Discussion

This investigation of diet in relation to symptoms associated with menopause was carried out among Bangladeshi women aged 35 to 59 years who were living in Bangladesh or who had migrated from Bangladesh to London as adults. We examined the self-reported frequency of hot flashes, night sweats, trouble sleeping, and vaginal dryness in relation to the estimated frequency of consumption of phytoestrogens. We hypothesized that diets rich in phytoestrogens (isoflavones, lignans, and coumestrol) would be associated with a lower frequency of symptoms.

With regard to hot flashes, the findings did not meet our expectations. After adjusting for menopausal status and parity, results from logistic regression showed that with each incremental increase in the phytoestrogen consumption range, the likelihood of hot flashes increased by 1.4%. The lignan consumption range included a subset of foods from the phytoestrogen consumption range, and with the more focused food list, each incremental increase in lignan consumption increased the likelihood of hot flashes by 1.6%. These relationships remained significant when the logistic regression models were limited to only sedentees in Bangladesh, and the direction of the relationships remained the same in models limited to only Bangladeshi migrants in London.

The findings with regard to vaginal dryness did meet our expectations in that the odds of vaginal dryness decreased with increasing phytoestrogen, lignan, and isoflavone consumption. These findings were for the sample as a whole (*n* = 211), and the direction of the relationships remained the same for sedentees (*n* = 95) and migrants (*n* = 116). The sample sizes were smaller for models of vaginal dryness because the symptom was considered a sensitive topic in this population and was not asked of every study participant.

Randomized controlled trials of phytoestrogen (usually soy) supplementation and symptoms at midlife have yielded mixed results, but there is general agreement that hot flash frequencies and severity decline as phytoestrogen intake increases [35,36]. A meta-analysis of clinical trials found that intake of phytoestrogens was associated with a decrease in both the number of daily hot flashes and the vaginal dryness score [36]. We showed the opposite findings for hot flashes, but our findings were consistent with regard to vaginal dryness. Lignan supplementation (usually flaxseed) has generally been less effective than isoflavone supplementation in reducing hot flashes [30]. In contrast, we found that the frequency of lignan intake was significantly associated with increased odds of hot flashes and decreased odds of vaginal dryness. The lack of a relationship between symptoms and the coumestrol consumption range is consistent with the lack of findings in supplementation studies [32]. In the meta-analysis of clinical trials mentioned above, the use of phytoestrogens was not associated with a change in the number of night sweats [36]. This is consistent with our findings which showed no relationship between night sweats and any measure of the frequency of phytoestrogen intake. Neither was there a relationship between trouble sleeping and the frequency of phytoestrogen intake after adjusting for covariates.

With regard to food patterns, the intake of vegetables and fruits has been inversely associated with hot flashes in some studies [40,41,42]. Our food list consisted primarily of vegetables, and we showed that both a general estimate of the frequency of phytoestrogen consumption and a more focused estimate of the frequency of lignan intake were associated with an increase in hot flash frequencies and a decline in vaginal dryness. It is not clear why the directions of the relationships differ, but future paths for investigation may include the estrogenic and antiestrogenic effects of phytoestrogens related to endogenous levels of estrogen, types of food eaten, combinations of food eaten, and variation in the gut microbiome [11,68,69], especially where populations are exposed to intestinal parasites and occasional prophylactic medicine targeting such parasites [44].

The relationship between phytoestrogens and hot flashes has been of interest for decades, prompted by the observation that Japanese women have consistently reported lower frequencies of hot flashes [3,4,5,6,7,8,9,10]. However, a study of hot flashes in Hawaii found that, although Japanese women were significantly less likely to report hot flashes by questionnaire, they did not differ in the frequency of objective hot flashes measured by skin conductance compared to their neighbors of European descent [70]. These findings suggest that lower frequencies of self-reported hot flashes among the Japanese may be a consequence of cultural conceptions rather than a higher intake of phytoestrogens. In the study presented here, we did not compare across ethnicities; however, our measurement of self-reported hot flashes may be assessing something unexpected related to cultural perceptions, which resulted in a reversal of the relationship between phytoestrogens and hot flashes. If this is the case, the cultural perceptions do not appear to differ between Bangladeshi migrants in London and Bangladeshi sedentees in Sylhet, Bangladesh, because the direction of the relationship between the increasing likelihood of hot flashes in relation to increasing phytoestrogen intake did not differ when each group was considered alone.

With regard to other results from the logistic regression models, peri-menopausal women, and postmenopausal women were more likely to report hot flashes and vaginal dryness, while postmenopausal status was associated with an increased likelihood of trouble sleeping. These findings are consistent with the literature [50]. The notable relationship between financial comfort and night sweats may be related to the physical environment in which women sleep. For example, women who are struggling financially may be less able to afford electric fans in Bangladesh, may have less access to electricity at night without private generators (having a private generator is common among the more affluent in Sylhet), and may have smaller, more crowded homes in London. The finding that Hindu women were more likely to report hot flashes is consistent with the portion of this same study that applied hot flash monitors to participants in Sylhet, Bangladesh. Hindu participants did not demonstrate more biometrically measured hot flashes, but they subjectively reported more hot flashes during the study period [64].

This study had several limitations. We estimated phytoestrogen intake instead of carrying out an actual measurement of intake. In addition, we asked for the frequency of consumption of individual foods, but we did not ask for the amounts eaten. In part, the lack of consensus about the relationship between phytoestrogens and menopausal symptoms is related to heterogeneity in study designs, dosage of consumed phytoestrogens, and a lack of control for the consumption of phytoestrogens from other sources [32,35]. The study presented here was not a randomized controlled trial. Instead, we queried foods normally eaten by Bangladeshis in order to estimate phytoestrogen intake in the overall diet. However, comparing the amounts of phytoestrogens in various foods is complicated by differences in food preparation and variation across phytoestrogen databases [1]. For the purposes of this study, we asked if food was most often consumed cooked or raw, and we sought phytoestrogen values accordingly. The phytoestrogen values provide information on relative intake. Finally, the self-report of symptoms is also a limitation of the study because self-reports are filtered through cultural expectations [70].

## 5. Conclusions

In summary, the relationship between the frequency of phytoestrogen consumption and hot flashes was the opposite of our expectations, but the relationship between the frequency of phytoestrogen intake and vaginal dryness upheld our expectations. Future directions for research in this population could include attention to variation in gut microbiomes, perhaps in relation to a history of parasites and infectious disease, which is relevant to many populations in low- and middle-income countries.

## Figures and Tables

**Table 1:** Estimated consumption frequency of foods among Bangladeshis in London and in Bangladesh from food list.

	Estimated Consumption Frequency Mean (s.d.) ^a^	Cooked or Raw	Phytoestrogens ^b^ µg/100 g	Isoflavones ^c^ µg/100 g	Lignans ^d^ µg/100 g	Coumestrol ^e^ µg/100 g
Tomatoes	7.4 (3.3) 56% daily	78% both raw/cooked	6	1	4	
Red lentils	5.6 (3.5) 47% weekly to daily	cooked	14	13	<1	0.00
Cauliflower	4.1 (3.0) 57% weekly to daily	cooked	12	<1	11	
Cucumber	3.7 (3.6) 53% weekly to daily	77% raw	13	<1	13	
Carrots	3.7 (3.3) 50% weekly to daily	50% cooked 42% both raw/cooked	114 (cooked) 125 (raw)	3 (cooked) 4 (raw)	111 (cooked) 121 (raw)	
Broad beans	3.7 (3.2) 50% weekly to daily	cooked	22	<1	21	0.00
Cabbage	3.6 (2.9) 52% weekly to daily	96% cooked	8	<1	7	
Pumpkin	2.1 (2.3) 48% monthly or occasionally	cooked	154	<1	154	
Dried hyacinth beans	2.0 (1.9) 10% once or twice/month	cooked				
Green peas	1.7 (2.3) 35% occasionally	92% cooked	1	1	<1	
Mung beans	1.5 (1.9) 33% occasionally		50	8	42	<1
Nuts and seeds	1.2 (2.0)	77% cooked	35	7	27	
Peanuts Almonds			131	18	112	
Cashew nuts	37% occasionally		122	22	99	
Broccoli	1.0 (1.8)	78% cooked	96	3	90	
Dried peas	0.9 (1.6) 51% occasionally	84% cooked	13	12	<1	Split peas, round 8.11
Chickpeas ^f^ Chickpea flour	0.8 (1.6)	85% cooked	420	416	4	Kala chana, type of chickpea 6.13
	1.2 (2.1)					
	53% occasionally					

^a^ Estimated average intake by Bangladeshis in Bangladesh and London across categories of 0 = never, 1 = occasionally, 2 = monthly, 3 = fortnightly, 4 = weekly, and 5 = daily. Most often selected category also given (e.g., daily, weekly, occasionally). ^b^ Most phytoestrogen, isoflavone, and lignan values from Kuhnle et al. (2009) [67]. Values for nuts and seeds from Thompson et al. (2006). ^c^ Isoflavones are the sum of daidzein, genistein, glycitein, biochanin A and formononetin (Kuhnle et al. 2009). ^d^ Lignans are the sum of secoisolariciresinol and matairesinol (Kuhnle et al. 2009). ^e^ U.S. Department of Agriculture—Iowa State University Database on the Isoflavone Content of Foods. 2007. https://www.ars.usda.gov/ARSUserFiles/80400525/data/isoflav/isoflav1-4.pdf, accessed on 1 September 2022. ^f^ Two varieties of chickpeas are used in the Bangladeshi diet, “dublee boot” (garbanzo beans) and “chola” (smaller brown chickpeas).

**Table 2:** Characteristics of Bangladeshi participants living in Bangladesh (sedentees) and London (migrants).

	Total *n* = 331 Mean (s.d.) or %	Sedentees *n* = 157 Mean (s.d.) or %	Migrants *n* = 174 Mean (s.d.) or %	*p*-Value Across Migrant Categories
Age at interview Mean (standard deviation)	46.6 (7.2)	46.8 (7.1)	46.4 (7.3)	
	Range	Range	Range	
	35.0–59.5	35.0–59.0	35.0–59.5	*p* = 0.634
Menopausal status (%)				
Pre-	52.4 (173)	49.7 (78)	54.9 (95)	
Peri-	7.0 (23)	6.4 (10)	7.5 (13)	
Post-	40.6 (134)	43.9 (69)	37.6 (65)	*p* = 0.494
Married (%)	97.1 (267)	95.7 (133)	98.5 (134)	*p* = 0.167
Parity (%)				
0	4.2 (14)	3.2 (5)	5.2 (9)	
1–2	28.1 (93)	38.2 (60)	19.0 (33)	
3–4	38.7 (128)	36.3 (57)	40.8 (71)	
5+	29.0 (96)	22.3 (35)	35.1 (61)	*p* < 0.001
Financial comfort (%)				
Struggling	25.5 (84)	11.0 (17)	38.5 (67)	
OK	37.4 (123)	38.1 (59)	36.8 (64)	
Comfortable	25.5 (84)	29.7 (46)	21.8 (38)	
Well-off	11.6 (38)	21.3 (33)	2.9 (5)	*p* < 0.001
Religion (%)				
Muslim	89.4 (294)	77.4 (120)	100 (174)	
Hindu	10.3 (34)	21.9 (34)	0	
Christian	0.3 (1)	0.6 (1)	0	*p* < 0.001
Smoke/use tobacco with betel nut (%)	38.2	40.5	36.3	*p* = 0.432
BMI kg/m^2^				
Mean (s.d.)	26.8 (3.7)	26.2 (4.1)	27.3 (3.2)	*p* = 0.006
Stress (scale of 1–6)				
Mean (s.d.)	4.3 (1.5)	3.9 (1.5)	4.6 (1.5)	*p* < 0.001
Phytoestrogen consumption range	41.9 (21.9)	58.1 (17.9)	29.0 (15.4)	
Mean (s.d.)	*n* = 292	*n* = 129	*n* = 163	*p* < 0.001
Lignan consumption range	35.7 (19.4)	49.3 (16.4)	24.7 (13.9)	
Mean (s.d.)	*n* = 295	*n* = 132	*n* = 163	*p* < 0.001
Isoflavone consumption range	13.5 (8.8)	18.4 (9.3)	9.3 (5.8)	
Mean (s.d.)	*n* = 311	*n* = 143	*n* = 168	*p* < 0.001
Coumestrol consumption range	1.7 (2.6)	2.4 (2.9)	1.1 (2.1)	
Mean (s.d.)	*n* = 321	*n* = 151	*n* = 170	*p* < 0.001

**Table 3:** Associations between symptom presence/absence and phytoestrogen consumption.

	Phytoestrogen Consumption Range Mean (s.d.)	Lignan Consumption Range Mean (s.d.)	Isoflavone Consumption Range Mean (s.d.)	Coumestrol Consumption Range Mean (s.d.)
Hot flashes				
Yes	44.6 (22.8) *n* = 132	38.3 (20.0) *n* = 133	13.5 (8.8) *n* = 139	1.9 (3.1) *n* = 142
No	39.5 (21.0) *n* = 159	33.4 (18.7) *n* = 161	13.4 (8.4) *n* = 171	1.5 (2.2) *n* = 178
	*p* = 0.050	*p* = 0.035	*p* = 0.950	*p* = 0.223
Night sweats				
Yes	41.2 (21.6) *n* = 91	35.2 (18.9) *n* = 91	12.5 (8.1) *n* = 94	1.6 (2.5) *n* = 97
No	42.1 (22.2) *n* = 200	35.9 (19.7) *n* = 203	13.9 (9.1) *n* = 216	1.7 (2.7) *n* = 223
	*p* = 0.754	*p* = 0.773	*p* = 0.216	*p* = 0.687
Trouble sleeping				
Yes	39.3 (20.6) *n* = 182	33.7 (18.2) *n* = 185	12.7 (8.2) *n* = 193	1.6 (2.6) *n* = 200
No	45.9 (23.7) *n* = 107	38.8 (21.3) *n* = 107	14.9 (9.7) *n* = 115	1.9 (2.7) *n* = 118
	*p* = 0.012	*p* = 0.029	*p* = 0.037	*p* = 0.397
Vaginal dryness				
Yes	39.7 (20.5) *n* = 77	33.8 (17.7) *n* = 78	12.4 (7.6) *n* = 85	1.8 (2.4) *n* = 88
No	45.7 (22.5) *n* = 135	39.2 (20.0) *n* = 137	15.0 (9.8) *n* = 141	2.1 (3.2) *n* = 146
	*p* = 0.055	*p* = 0.048	*p* = 0.034	*p* = 0.438

**Table 4:** Logistic regression results for hot flashes in two separate models including phytoestrogen and lignan consumption.

	Total Sample OR (95% CI)	Migrants in London OR (95% CI)	Sedentees in Bangladesh OR (95% CI)
Model with phytoestrogen consumption			
	*n* = 290	*n* = 162	*n* = 128
Menopausal status			
Pre- (ref)			
Peri-	4.02 (1.45–11.14)	6.22 (1.56–24.79)	2.34 (0.49–11.24)
Post-	1.70 (1.01–2.87)	2.55 (1.22–5.33)	1.25 (0.55–2.84)
Parity	1.11 (0.96–1.28)	1.05 (0.87–1.28)	1.16 (0.92–1.45)
Phytoestrogen consumption range	1.014 (1.002–1.025)	1.014 (0.991–1.039)	1.028 (1.006–1.050)
Model with lignan consumption			
	*n* = 293	*n* = 162	*n* = 131
Menopausal status			
Pre-(ref)			
Peri-	4.02 (1.45–11.13)	6.11 (1.16–27.41)	2.45 (0.51–11.79)
Post-	1.67 (0.99–2.81)	2.58 (2.49–17.41)	1.19 (0.53–2.68)
Parity	1.11 (0.96–1.28)	1.058 (0.87–1.28)	1.15 (0.92–1.44)
Lignan consumption range	1.016 (1.004–1.029)	1.02 (0.993–1.045)	1.029 (1.006–1.054)
Isoflavone consumption not significant in relation to hot flashes.			

**Table 5:** Logistic regression results for night sweats in two separate models including phytoestrogen and lignan consumption.

	Total Sample OR (95% CI)	Migrants in London OR (95% CI)	Sedentees in Bangladesh OR (95% CI)
Model with phytoestrogen consumption			
	*n* = 275	*n* = 158	*n* = 117
Menopausal status			
Pre- (ref)			
Peri-	1.83 (0.67–5.04)	4.53 (1.23–16.67)	0.16 (0.02–1.68)
Post-	1.23 (0.67–2.27)	2.29 (1.01–5.23)	0.53 (0.18–1.53)
Parity	1.17 (1.02–1.43)	1.07 (0.86–1.33)	1.48 (1.11–1.99)
Financial comfort			
Struggling (ref)			
OK	0.43 (0.21–0.85)	0.42 (0.19–0.94)	0.62 (0.12–3.11)
Comfortable	0.36 (0.17–0.78)	0.20 (0.07–0.61)	0.83 (0.17–4.02)
Well-off	0.19 (0.06–0.59)	--	0.40 (0.07–2.35)
Religion			
Muslim (ref)			
Hindu	2.92 (1.04–8.24)	-- ^a^	4.17 (1.26–13.84)
Smoke/Tobacco with betel nut			
No (ref)			
Yes	1.57 (0.66–2.03)	0.95 (0.44–2.05)	1.50 (0.59–3.80)
Phytoestrogen consumption range	1.001 (0.986–1.017)	1.009 (0.984–1.035)	1.002 (0.973–1.031)
Model with lignan consumption			
	*n* = 278	*n* = 158	*n* = 120
Menopausal status			
Pre- (ref)			
Peri-	1.85 (0.67–5.10)	4.44 (1.21–16.33)	0.17 (0.02–1.76)
Post-	1.26 (0.69–2.32)	2.34 (1.03–5.32)	0.54 (0.19–1.56)
Parity	1.21 (1.02–1.44)	1.07 (0.86–1.33)	1.50 (1.12–2.01)
Financial comfort			
Struggling (ref)			
OK	0.42 (0.21–0.83)	0.41 (0.18–0.93)	0.59 (0.12–2.97)
Comfortable	0.35 (0.16–0.77)	0.20 (0.06–0.60)	0.81 (0.17–3.95)
Well-off	0.18 (0.06–0.56)	--	0.38 (0.07–2.28)
Religion			
Muslim (ref)			
Hindu	2.64 (0.96–7.28)	-- ^a^	4.21 (1.29–13.76)
Smoke/Tobacco with betel nut			
No (ref)			
Yes	1.18 (0.67–2.08)	0.97 (0.45–2.09)	1.60 (0.63–4.02)
Lignan consumption range	1.003 (0.986–1.020)	1.014 (0.985–1.043)	0.999 (0.970–1.030)
Isoflavone consumption not significant in relation to night sweats.			

^a^ All migrants were Muslim.

**Table 6:** Logistic regression results for trouble sleeping in two separate models including phytoestrogen and lignan consumption.

	Total Sample OR (95% CI)	Migrants in London OR (95% CI)	Sedentees in Bangladesh OR (95% CI)
Model with phytoestrogen consumption			
	*n* = 264	*n* = 151	*n* = 113
Menopausal status			
Pre- (ref)			
Peri-	1.53 (0.52–4.49)	3.43 (0.64–18.35)	0.40 (0.05–3.00)
Post-	2.33 (1.25–4.35)	3.22 (1.26–8.21)	1.87 (0.68–5.15)
Parity	1.01 (0.85–1.20)	1.15 (0.89–1.49)	0.95 (0.72–1.26)
Financial comfort			
Struggling (ref)			
OK	0.39 (0.18–0.87)	0.63 (0.25–1.61)	0.08 (0.01–0.79)
Comfortable	0.53 (0.22–1.27)	0.50 (0.17–1.48)	0.22 (0.02–2.25)
Well-off	0.46 (0.16–1.30)	0.38 (0.05–3.12)	0.15 (0.01–1.65)
Smoke/Tobacco with betel nut			
No (ref)			
Yes	2.55 (1.42–4.60)	2.18 (0.92–5.16)	2.86 (1.15–7.11)
BMI	1.10 (1.02–1.19)	0.99 (0.86–1.13)	1.16 (1.04–1.30)
Stress	1.21 (1.00–1.47)	1.41 (1.05–1.89)	1.23 (0.90–1.67)
Phytoestrogen consumption range	0.996 (0.983–1.009)	1.003 (0.977–1.030)	0.988 (0.964–1.013)
Model with lignan consumption			
	*n* = 266	*n* = 151	*n*= 115
Menopausal status			
Pre-(ref)			
Peri-	1.50 (0.51–4.42)	3.44 (0.64–18.41)	0.41 (0.55–3.10)
Post-	2.35 (1.26–4.38)	3.21 (1.26–8.16)	2.00 (0.74–5.44)
Parity	1.01 (0.85–1.20)	1.15 (0.89–1.49)	0.94 (0.71–1.24)
Financial comfort			
Struggling (ref)			
OK	0.39 (0.18–0.85)	0.63 (0.25–1.61)	0.08 (0.01–0.79)
Comfortable	0.53 (0.22–1.27)	0.50 (0.17–1.48)	0.23 (0.02–2.39)
Well-off	0.45 (0.16–1.27)	0.38 (0.05–3.12)	0.16 (0.01–1.74)
Smoke/Tobacco with betel nut			
No (ref)			
Yes	2.47 (1.37–4.44)	2.19 (0.93–5.17)	2.61 (1.06–6.43)
BMI	1.11 (1.03–1.20)	0.99 (0.86–1.13)	1.17 (1.04–1.30)
Stress	1.21 (1.00–1.47)	1.41 (1.05–1.89)	1.21 (0.89–1.64)
Lignan consumption range	0.998 (0.983–1.013)	1.003 (0.975–1.033)	0.994 (0.968–1.020)
Isoflavone consumption not significant in relation to trouble sleeping.			

**Table 7:** Logistic regression results for vaginal dryness in three separate models including phytoestrogen, lignan, and isoflavone consumption.

	Total Sample OR (95% CI)	Migrants in London OR (95% CI)	Sedentees in Bangladesh OR (95% CI)
Model with phytoestrogen consumption			
	*n* = 211	*n* = 116	*n* = 95
Menopausal status			
Pre-(ref)			
Peri-	3.69 (1.17–11.67)	5.48 (1.13–26.57)	2.25 (0.36–14.29)
Post-	5.49 (2.85–10.57)	6.43 (2.44–16.98)	5.09 (1.93–13.41)
Phytoestrogen consumption range	0.984 (0.970–0.998)	0.981 (0.954–1.009)	0.993 (0.967–1.019)
Model with lignan consumption			
	*n* = 214	*n* = 116	*n* = 98
Menopausal status			
Pre-(ref)			
Peri-	3.75 (1.19–11.82)	5.63 (1.16–27.41)	2.34 (0.37–14.86)
Post-	5.74 (2.99–11.02)	6.59 (2.49–17.41)	5.59 (2.13–14.64)
Lignan consumption range	0.982 (0.966–0.998)	0.978 (0.948–1.008)	0.992 (0.965–1.021)
Model with isoflavone consumption			
	*n* = 225	*n* = 118	*n* = 107
Menopausal status			
Pre-(ref)			
Peri-	3.14 (1.01–9.78)	5.22 (1.08–25.28)	1.63 (0.27–10.01)
Post-	4.64 (2.50–8.60)	6.18 (2.36–16.24)	3.72 (1.56–8.90)
Isoflavone consumption range	0.963 (0.930–0.998)	0.956 (0.890–1.026)	0.972 (0.925–1.021)

## Data Availability

Data presented in this study are available on request from the corresponding author. Data will be available as part of a merged dataset of studies of menopause in eight countries through ScholarWorks@UMass Amherst, a digital repository of research and scholarship.

## Supplementary Materials

The following supporting information can be downloaded at: https://www.mdpi.com/article/10.3390/nu15173676/s1, Table S1: Percentage of women with hot flashes, night sweats, trouble sleeping, and vaginal dryness by sample characteristics (Chi-square analyses); Table S2: Presence/absence of symptoms in relation to BMI and stress (*t*-tests).
