# Supplementary material for: Frequency of Phytoestrogen Consumption and Symptoms at Midlife among Bangladeshis in Bangladesh and London
Source: Nutrients. 2023 Aug 22;15(17):3676. doi: 10.3390/nu15173676 (PMC10490262; doi:10.3390/nu15173676)
Supplement: Supplementary file 1 [file nutrients-15-03676-s001.zip › nutrients-2537500-supplementary.pdf]

Table S1. Percentage of women with hot flashes, night sweats, trouble sleeping, and vaginal dryness by sample characteristics (Chi-square analyses)[a].

	Hot flashes (%) n=331	Night sweats (%) n=330	Trouble sleeping (%) n=328	Vaginal dryness (%) n=243
Migrant status				
Sedentees	45.5	26.1	59.7	36.1
Migrants	43.1	34.1	65.5	37.9
	p=0.660	p=0.115	p=0.280	p=0.775
Age category	**25.7**	**18.3**	**45.7**	**13.8**
35-39 years	**36.6**	**28.2**	**60.6**	**30.0**
40-44 years	**61.5**	**39.1**	**66.2**	**49.1**
45-49 years	**67.2**	**43.8**	**77.8**	**58.8**
50-54 years	**31.7**	**23.3**	**66.1**	**54.8**
55-59 years	**p<0.001**	**p=0.007**	**p=0.004**	**p<0.001**
Menopausal status				
Pre-	**36.6**	**25.0**	**55.2**	**24.3**
Peri-	**73.9**	**47.8**	**69.6**	**46.7**
Post-	**49.3**	**34.3**	**72.0**	**61.3**
	**p=0.001**	**p=0.036**	**p<0.009**	**p<0.001**
Married	50.0	28.6	62.5	50.0
No	43.2	29.6	60.0	36.9
Yes	p=0.704	p=0.954	p=0.887	p=0.592
Parity				
0	**42.9**	**50.0**	**50.0**	66.7
1-2	**33.7**	**15.1**	**56.5**	27.6
3-4	**44.5**	**29.9**	**59.1**	40.6
5+	**54.2**	**42.7**	**75.8**	38.6
	**p=0.046**	**p<0.001**	**p=0.017**	p=0.076
Financial comfort				
Struggling	52.4	**47.6**	**75.9**	40.4
OK	41.5	**25.4**	**58.5**	44.1
Comfortable	44.6	**25.0**	**57.3**	26.7
Well off	34.2	**18.4**	**57.9**	32.3
	p=0.241	**p<0.001**	**p=0.037**	p=0.152
Religion	42.7	**28.3**	62.7	36.5
Muslim	58.5	**47.1**	66.7	40.9
Hindu	p=0.073	**p=0.024**	p=0.652	p=0.685
Smoke or tobacco with betel nut				
No	41.1	**25.0**	**55.6**	33.1
Yes	48.8	**36.9**	**75.0**	41.3
	p=0.182	**p=0.024**	**p<0.001**	0.220

[a] Bolded results indicate sample characteristics included in logistic regressions for individual symptoms.

Table S2. Presence/absence of symptoms in relation to BMI and stress (T-tests)[a].

	BMI<br>Mean (s.d.)	Stress<br>(scaled 1-6)<br>Mean (s.d.)
Hot flashes		
Yes	27.2 (3.8)	4.3 (1.4)
No	26.5 (3.6)	4.2 (1.5)
	p=0.112	p=0.127
Night sweats		
Yes	27.1 (3.7)	4.4 (1.4)
No	26.6 (3.7)	4.2 (1.5)
	p=0.241	p=0.185
Trouble sleeping		
Yes	**27.3 (3.8)**	**4.4 (1.4)**
No	**25.9 (3.4)**	**4.0 (1.6)**
	**p=0.001**	**p=0.011**
Vaginal dryness		
Yes	26.6 (3.5)	4.4 (1.3)
No	26.7 (3.7)	4.2 (1.5)
	p=0.838	p=0.201

[a] Bolded results indicate sample characteristics included in logistic regressions for individual symptoms.
